# HOW PERSON-CENTERED SERVICE DELIVERY SUPPORT QUALITY OF LIFE: WHAT NCI-AD TELLS US

**DOI:** 10.1093/geroni/igad104.2919

**Published:** 2023-12-21

**Authors:** Stephanie Giordano, Rosa Plasencia

**Affiliations:** Human Services Research Institute, Cambridge, Massachusetts, United States; ADvancing States, Arlington, Virginia, United States

## Abstract

COVID-19 left a broad impact - for many older adults the pandemic led to changes in perceptions of person-centered services and experience with LTSS. Person-centered service delivery is widely accepted as a gold standard to supporting better outcomes, experience, and satisfaction; yet, there remains a gap in understanding person-centeredness from the perspective of people receiving the service. NCI-AD surveyors hear directly from older adults and people with physical disabilities on satisfaction with service delivery and quality of life. Using 2021-22 data, we’ll discuss the perception of responsiveness and person-centered service delivery among other important measures. For example, older adults who were satisfied with provider response were more likely than those who were not satisfied to have more positive outcomes related to access to the community, ability to maintain relationships, and fewer unmet service needs. This session will delve further into this relationship between person-centered service delivery and quality of life outcomes, addressing what this means for policy and practice. Data presented come from the 2021-2022 National Core Indicators® Aging and Disabilities (NCI-AD™) Adult Consumer Survey (ACS). The ACS includes two main survey sections. The Background Information provides key data about survey participants, including demographics, service-related characteristics, and clinical diagnoses, typically collected from administrative records about the person. The main survey section includes questions on a range of topics that address the quality of the service delivery system; including responsiveness of services and supports, availability of needed services, and quality of life outcomes.

